# The role of BAFF and G-CSF for rituximab-induced late-onset neutropenia (LON) in lymphomas

**DOI:** 10.1007/s12032-021-01516-8

**Published:** 2021-05-18

**Authors:** Daniel Tesfa, Birgitta Sander, Henric Lindkvist, Christer Nilsson, Eva Kimby, Hans Hägglund, Björn E. Wahlin, Monika Klimkowska, Jan Palmblad

**Affiliations:** 1 The Hematology Center and Department of Medicine, Karolinska Institutet, Karolinska University Hospital Huddinge R51, 141 86 Stockholm, Sweden; 2 Department of Clinical Pathology and Cytology, Department of Laboratory Medicine, Karolinska University Hospital Huddinge, Karolinska Institutet, 141 86 Stockholm, Sweden; 3 Present Address: Swedish Orphan Biovitrum AB, 112 76 Stockholm, Sweden; 4 Division of Hematology, Department of Medical Sciences, Uppsala University, Uppsala, Sweden

**Keywords:** Late-onset neutropenia, Rituximab, G-CSF, BAFF, APRIL, SDF1

## Abstract

Mechanisms for late-onset neutropenia (LON) after rituximab treatment are poorly defined both for non-Hodgkin lymphoma (NHL) and for autoimmune disorders. We performed a case–control analysis of a prospective cohort of 169 evaluable consecutive rituximab-treated NHL patients to assess cytokines involved in neutro- and lymphopoiesis (G-CSF, SDF1, BAFF, APRIL) and inflammation (CRP) as possible LON mechanisms. Fifteen patients (9%) developed LON (peripheral blood /PB/ absolute neutrophil counts /ANC/ < 0.5 G/L, all with marked depletion of CD20^+^ B-lymphocytes in bone marrows); they were compared with 20 matched NHL controls without LON. At start of LON, significantly higher PB G-CSF and BAFF levels (*P* = 0.0004 and 0.006, respectively), as well as CRP rises were noted compared to controls; these G-CSF and BAFF and most CRP values returned to levels of the controls in post-LON samples. G-CSF (but not BAFF) changes correlated to CRP rises (but not to ANC levels). BAFF levels correlated significantly to absolute monocyte counts and PB large granular lymphocyte counts (but not to ANC, C-CSF or CRP values). No changes of SDF1 or APRIL levels were noted. Neither LON cases nor controls displayed anti-neutrophil autoantibodies. Collectively, LON in NHL patients was timewise related to transient bursts of blood G-CSF and BAFF concentrations, suggesting that these neutro- and lymphopoiesis growth factors play a role in emergence of rituximab-induced LON, and that inflammation may be a trigger for G-CSF production during LON.

**Supplementary Information:**

The online version contains supplementary material available at 10.1007/s12032-021-01516-8.

## Introduction

Late-onset neutropenia (LON), following rituximab therapy for autoimmune diseases or lymphomas, is an unusual type of drug-induced neutropenia (NP), because it occurs between 1 and 12 months after completing rituximab therapy (and not during or shortly after intake as with most agranulocytosis-inducing drugs) [1–3]. The mechanisms for LON remain, however, poorly defined [2, 3].

Recently, the role of BAFF (B-cell activating factor) has attracted attention in relation to rituximab treatment for rheumatic diseases [4–7]. Neutrophils and monocytes are important sources of BAFF and its release is induced by, for example, granulocyte colony-stimulating factor (G-CSF) [8–10]. BAFF, a member of the TNF family, plays a central role in the stimulation of B-lymphocytes and is a survival factor for transitional and mature B cells and immunoglobulin production [10]. High levels of BAFF may also induce autoantibodies and enhance the rheumatic process [5, 7].

After rituximab therapy, CD20^+^ B-lymphocytes are rapidly depleted and they reappear to the peripheral blood (PB) after 5–12 months [6, 11, 12], preceded by an elevation of peripheral blood (PB) levels of BAFF [4].Elevated BAFF levels have also been reported in LON in rheumatic diseases, with a return to basal levels after LON [6], yet its role in LON development is unclear, as well as the underlying mechanisms involved in BAFF elevation. For instance, concomitant infection/ inflammation involving transient G-CSF production may promote BAFF release, as shown in other settings [8–10]. In addition, we and others reported that LON in lymphoma and rheumatic patients is related to the possession of certain *BAFF* and *FCGR3* gene polymorphisms [13, 14], suggesting possible gene-drug interactions.

To assess if there is a relationship between emergence of LON in NHL patients and coexistent perturbations of BAFF production, we performed a case–control analysis of a prospective cohort of 174 consecutive NHL patients treated with rituximab. We used PB and bone marrow (BM) samples obtained at the time of detection of LON and in post-LON samples. We also analyzed G-CSF (a BAFF-promoting cytokine [8, 10], also central for emergency neutropoiesis [9, 15]) and C-reactive protein (CRP, as a sign of inflammation/infection). Further, we studied APRIL (A Proliferation-Inducing Ligand, also secreted by myeloid and other cells, being a proliferation and maturation factor for B-lymphocytes [6, 9, 16], as well as stromal-derived factor-1 (SDF-1/CXCL12), a molecule involved in neutrophil egress from and return to the BM [11, 17]. Finally, we analyzed the presence of anti-neutrophil autoantibodies and large granular lymphocytes, LGL (as consequences of autoimmunity-promoting BAFF rises [5, 7] and being central for development of autoimmune NPs), shifts of T-lymphocyte subpopulations and NK cell numbers in PB and BM. Thus, LON was used as a model to analyze possible mechanisms for a drug-induced neutropenia (DINP).

## Patients and methods

### Study design and patients

#### Inclusions

The study, approved by the Ethics committee at Karolinska Institutet, Stockholm, Sweden, was performed in accordance with the Helsinki declaration. All patients gave their written informed consent before initiation of rituximab treatment.

**Fig. 1:**
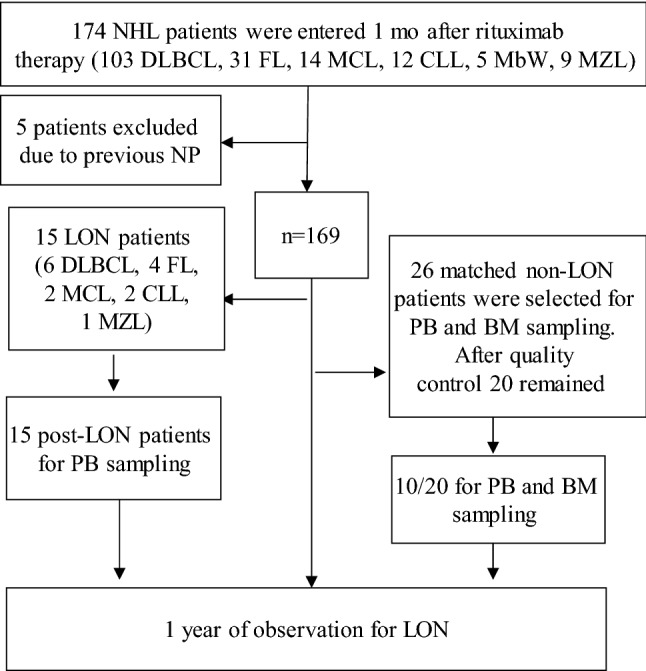
Consort flow diagram of the study. *LON* late onset neutropenia, *NP* neutropenia, *DLBCL* diffuse large B-cell lymphoma, *FL* follicular lymphoma, *MCL* mantle cell lymphoma, *CLL* chronic lymphocytic leukemia, *MbW* Mb Waldenström (macroglobulinemia), *MZL* marginal zone lymphoma

We included prospectively 174 consecutive adult NHL patients, treated with rituximab at the Hematology Center, Karolinska University Hospital, Huddinge, Stockholm, Sweden. Figure 1 summarizes the design and essentials of the study.

#### Patients

Patients were treated according to standard care protocol at the discretion of the treating physician; however, all received rituximab. Median age was 62 years (range 26–83). Ninety were males and 79 females. Seventeen patients were treated with rituximab alone, at 375 mg/m^2^. The others received rituximab, at the same dose, in combination with chemotherapy; details are given in Table 1. CBCs (complete blood counts) and PB C-reactive protein (CRP) levels were obtained routinely every fortnight to month.

**Table 1:** Demographic characteristics and treatments of the LON and non-LON NHL control patients

	LON-patients	Controls	*P*-value
	*n* = 15	*n* = 26	
Age, years, median ± SD (min–max)	58 ± 11.8 (35–74)	61.5 ± 15,9 (27–86)	0.6
Sex, *n* (%)			
Males	8 (53%)	14(54%)	0.8
Females	7 (47%)	12(46%)	0.8
Ann Arbor stage at diagnosis, *n* (%)			
I–II	2 (13%)	6 (23%)	0.8
III–IV	13 (87%)	20 (77%)	0.8
Treatment type and intensity, *n* (%)			
De novo treatment	8 (57%)	16 (62%)	0.8
Previous rituximab therapy	6 (43%)	9 (35%)	0.8
Rituximab maintenance therapy	1 (7%)	2 (8%) 1.0	
Relapse treatment	6 (43%)	10 (38%)	0.8
Antracycline based treatment	8 (57%)	18 (69%)	0.6
Fludarabine based treatment	2 (14%)	4 (15%)	0.9
HSCT treatment	5 (36%)	8 (31%)	0.7
Other treatment modalities	4 (29%)	4 (15%)	0.6
Methotrexate (HDMTX) dose, mg, mean ± SD	5200 ± 848.5	5300 ± 953.9	0.18
Rituximab dose, mg, mean ± SD	3850 ± 598.4	4253.9 ± 738.8	0.15

*SD* standard deviation; *IQR* interquartile range; *HSCT* autologous hematological stem cell transplantation; *HDMTX* high-dose methotrexate

#### Exclusions

Medical records were reviewed for the time of two years before the start of rituximab therapy and all patients with a previous history of NP were excluded. Hence, 169 patients were included for LON analysis (Fig. 1 and Supplementary Material).

#### Definition of LON

LON is defined as an otherwise unexplained PB absolute neutrophil count (ANC) of ≤ 0.5 G/L (corresponding to grade 4 neutropenia according to National Cancer Institute Common Toxicity Criteria (NCI-CTC) [18], and agranulocytosis as ANC ≤ 0.1 G/L, starting earliest four weeks after the termination of rituximab therapy. Absolute PB monocyte counts (AMC) were considered normal if 0.2–0.8 G/L. All patients were followed for, at least, 12 months after rituximab treatment [19] (Supplementary Material).

#### Controls

A control group within the same cohort of NHL patients was established simultaneously with detection of a LON episode. This comparison was done to control for confounding factors for the NP. See Supplementary Material for details. In the end, 26 control subjects were included (Table 1). Full sets of PB samples were obtained from 20 of these controls. In addition, a subgroup (*n* = 10) of these 20 patients volunteered to also provide BM samples.

### Peripheral blood (PB) and bone marrow (BM) evaluations

In LON patients, PB samples were obtained twice: first, at detection of LON (“LON samples”) and, second, after resolution of LON (“post-LON samples”). BM samples were obtained at the first PB sampling. We also retrieved clinical PB ANC and AMC immediately preceding onset of a LON episode (i.e.1/–2/ weeks). In patients given G-CSF because of LON, BM and PB samples were collected before start of G-CSF treatment (except for one case) and, at least, one week after stopping that therapy (except for two cases); all data shown are for those without ongoing or recent G-CSF treatment. LON and post-LON PB samples were also used for serology and serum cytokine tests and CRP (analyzed according to hospital routines). See also Supplementary Material.

Relative LGL cell abundance was analyzed by light microscopy on routinely stained PB smears, as advised by Loughran and Lamy [20].

In the matched controls, BM (*n* = 10) and PB samples (*n* = 20) were also collected and processed at time points corresponding to the detection of LON in the LON patients, to control for potential time-related effects for BM regeneration after the last rituximab infusion and chemotherapy.

*BM flow cytometry* was performed by fluorescence immunophenotyping for B and T lymphocytes, and for natural killer (NK) cells, as described [14, 19]. For analysis of B-cells, fluorochrome-conjugated monoclonal antibodies to B-cells antigens CD19 and CD20 were used. For analysis of T-cells and NK-cells, anti-CD3, anti-CD4, anti-CD8, anti-CD16 and anti-CD56 antibodies were used. See also Supplementary Material.

B cell depletion (analyzed by flow cytometry) was analyzed as percentage of BM cells expressing CD20.

### Enzyme linked immunosorbent assay (ELISA)

Serum levels of human BAFF, APRIL, G-CSF and plasma levels of SDF-1 (a k a CXCL12) were analyzed using Quantikine immunoassays (R&D Systems Europe) according to the manufacturer’s instructions.

### Anti-neutrophil antibodies

The test panel included granulocyte agglutination test (GAT) and granulocyte immunofluorescence tests (GIFT), followed by monoclonal antibody-specific immobilization of granulocyte antigen (MAIGA) test [21]. See Supplementary Material for details.

### Statistical analyses

Values are presented as mean ± SD or median (and interquartile range, IQR) depending on value distributions. The chi-squared test and Fisher's exact test were used to compare patient characteristics, treatments and proportions. Wilcoxon’s matched pairs/signed rank test was performed to assess differences in lymphocyte subpopulations at different times for paired samples of LON and non-LON-matched control patients. Associations between time to LON/duration of LON, ANC/AMC, cytokine, CRP levels and ANC were analysed by Spearman’s correlation analysis. All tests were 2-sided and P values less than 0.05 were considered significant.

## Results

### LON characteristics

Fifteen patients (8.8% of all patients in the cohort) presented with LON (eight males and seven females, median age 58 years; Fig. 1). LON patients and their 26 matched non-LON controls had similar demographics, NHL subdiagnoses and treatments (including rituximab and methotrexate) as the other NHL patients (Table 1 and Supplementary Table 1).

After initial lymphoma treatment, all LON and control patients had recovered normal ANCs, were in clinical remission and had no other identifiable causes of NP than LON. Post-LON PB samples were obtained from all 15 LON patients (still being in remission) within 3 weeks after LON resolution. In the end, blood samples from 20 non-LON controls (out of the initially selected 26) remained because of inappropriate collection and assaying.

**Fig. 2:**
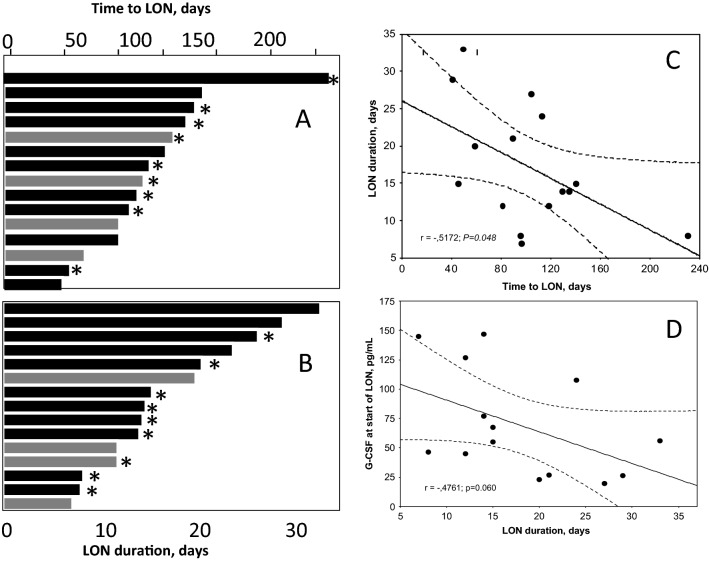
Time to LON (panel **A**), duration of LON (panel **B**), correlation between time to and duration of LON (panel **C**) and correlation between PB levels of G-CSF and duration of LON (panel **D**). Grey bars in panel **A** represent patients with a previous HSCT. Asterisks denote patients treated with filgrastim/i.v. antibiotics during LON

*The characteristics of LON patients* are given in detail in Supplementary material. Briefly, the median time to onset of LON was 96 days and median duration of LON was 17 days (Figs. 2A, B). Four patients were febrile at LON detection and had moderate CRP rises (< 59, reference value < 4 mg/L); in addition, three more had minor CRP rises (< 20). Nine patients received short term treatment with G-CSF (Fig. 2A, B).

**Fig. 3:**
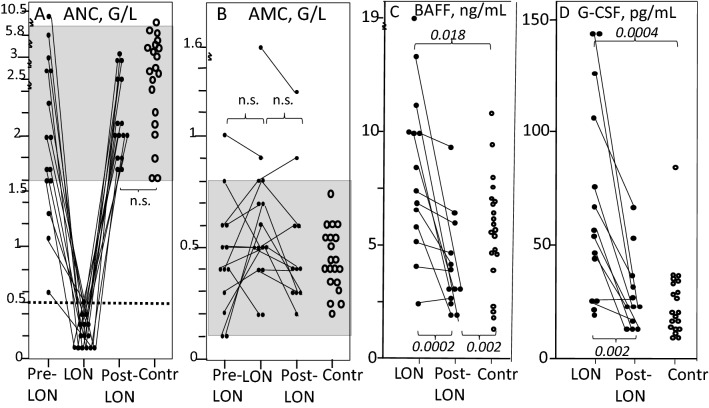
Peripheral blood (PB) absolute neutrophil counts (ANC, panel **A**), and absolute monocyte counts (AMC, panel **B**). Panel **C** shows PB BAFF levels and panel **D** PB G-CSF values. Grey areas represent reference values for healthy controls. *P*-values are given close to brackets. *n.s.* no significant differences. Solid circles = LON patients, open circles = matched non-LON patients. Pre-LON samples are those obtained immediately prior to the start of LON, LON = at diagnosis of LON, and post-LON = upon recovery from LON

*The ANC*. The median nadir ANC was 0.2 G/L; thus, all LON patients developed severe NP (Fig. 3A). None of the 20 non-LON controls with available CBC, corresponding to the time to LON of their matched pairs, displayed ANC < 1.5 G/L (*P* < 0.0001; Fig. 3A).

*The AMC* for LON patients were similar to those of non-LON controls (*P* > 0.05 for all comparisons; Fig. 3B) and did not correlate to change of ANCs (*P* > 0.05); thus, those with the most pronounced ANC drop did not raise their AMC more than those with the least pronounced ANC drops.

*Time to LON and LON duration* correlated significantly, in that those with a short time to LON had longer duration of LON than those with a long time to LON (*P* = 0.048; Fig. 2C). LON-patients with a previous HSCT (*n* = 4; 27% of all LON patients) had similar incidences and complications to LON as those without (Fig. 2A, B).

*Post-LON data.* At time for post-LON sampling, all LON patients had regained normal ANCs, AMCs remained unchanged, raised CRP values had returned to normal in 3/5 and remained moderately elevated in 2/5.

**Fig. 4:**
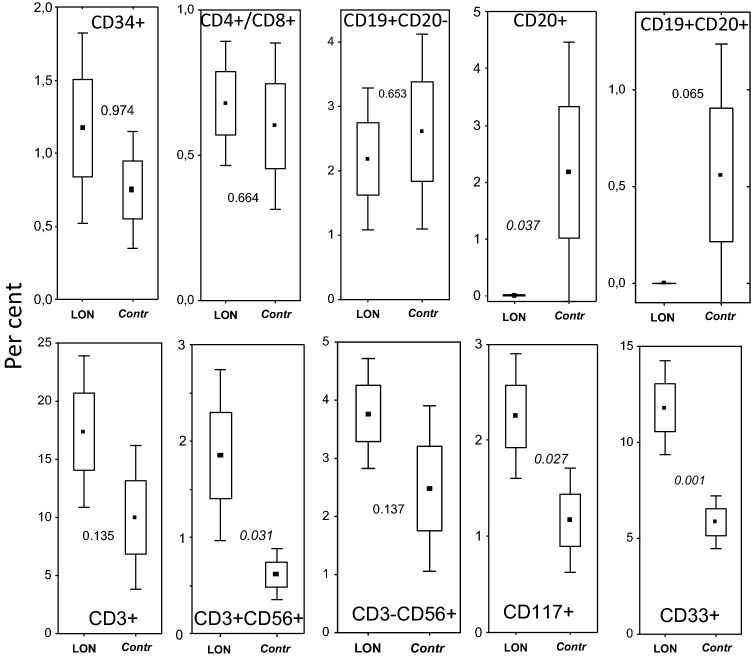
Flow cytometry analysis of BM cells. Results are given as percent of cells in the mononuclear gate, except for the CD4/CD8 ratio. *P*-values are given between the boxes. Mean (filled squares), SE (boxes) and 1.96*SE (whiskers) values. Statistical significance (*p* < 0.05) between LON and controls is indicated by italic figures

*B-lymphocyte depletion at start of LON.* Only LON-patients displayed a complete depletion of BM CD20^+^cells (Fig. 4), whereas LON-patients and non-LON controls did not differ in numbers of CD19^+^CD20^−^ B-lymphocytes in the BM, as analysed by flow cytometry.

*BM lymphocyte subpopulations, myeloid, NK and LGL cells and relation to LON.* The numbers of CD3^+^ (T-cells) and CD3^−^CD56^+^cells (NK cells) as well as numbers of CD4^+^or CD8^+^ cells or in CD4^+^/CD8^+^ ratio (i.e. subpopulations of T-cells) did not differ significantly between the LON or control groups (Fig. 4). However, we did observe significantly higher numbers of CD3^+^CD56^+^ (T/NK-cells) in LON than in control patients (Fig. 4).

Percentages of CD33^+^ and CD117^+^ cells were significantly higher in LON patients at start of LON than in controls (Fig. 4).

No significant associations were noted for relations of CD3^+^56^+^ cells to ANC and AMC (data not shown).

*LGL* cells were found in PB samples in LON patients trendwise more often than in controls (14.3% of all lymphocytes vs 9.2%, respectively; *P* = 0.057). The percentages of LON patients did not correlate to numbers of CD3^+^CD56^+^ (T/NK-cells; *P* > 0.05).

### Serum BAFF levels

Previously, we showed that B-lymphocyte depletion coincided timewise with LON, and regenerated B-lymphocyte numbers with normalized ANC; this suggested that LON may be related to factors for proliferation of B-lymphocytes, such as BAFF [6, 14, 17, 19].

At onset of LON, serum BAFF values were significantly higher in LON patients compared to non-LON controls (*p* = 0.018). In post-LON samples, BAFF levels decreased (*p* = 0.0002 for the difference between LON and post-LON samples), and were then significantly lower than in non-LON controls (*p* = 0.002) (Fig. 3C).

**Fig. 5:**
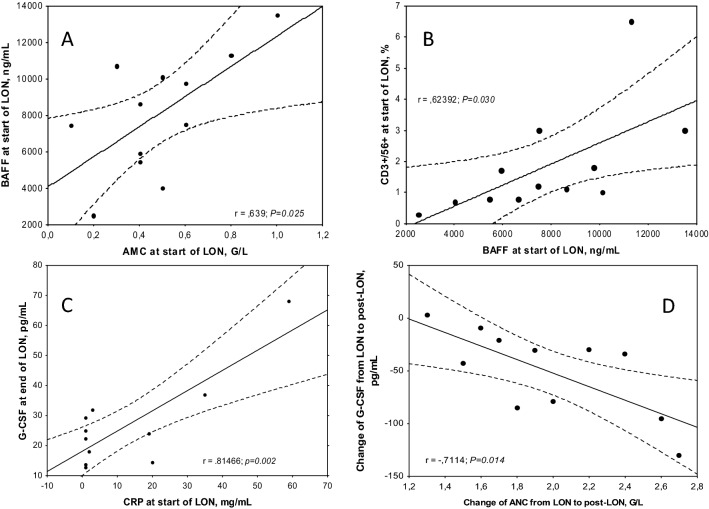
Spearman’s correlations between: Panel **A** peripheral blood absolute monocyte counts (AMC) and BAFF concentrations, both at start of LON; Panel **B** BAFF concentrations and CD3 + CD56 + cells in the bone marrow, both at start of LON; Panel **C** concentrations of CRP at start of LON and G-CSF at end of LON; and Panel **D** change of absolute neutrophil counts (ANC) and G-CSF concentrations, both as the change between start of LON and post-LON values. *P*-values are given after the *r*-values. Significand correlations are marked in italics

Considering that BAFF is produced to a large extent by monocytes and neutrophils [8–10, 15] and that the LON patients were severely neutropenic, but not monocytopenic at LON onset, we asked if the AMC correlated with BAFF levels at onset of LON and we found a significant correlation (*r* = 0.639, *P* = 0.025; Fig. 5A). There were no significant correlations between BAFF levels at onset of LON and the ANC, CD33^+^ or CD117^+^ cells at this time, neither was there a significant correlation of BAFF changes and the ANCs over the LON period (data not shown). Furthermore, BAFF levels did not correlate with CRP levels at start of LON or with time to or duration of LON (*P* > 0.05; data not shown). However, BAFF figures correlated positively significantly to numbers of CD3^+^CD56^+^ (T/NK-cells; *P* = 0.030; Fig. 5B). Thus, positive relations of BAFF levels to PB monocyte and CD3^+^CD56^+^ (T/NK-cells) counts were found, but not to signs of inflammation (CRP) or of NP (i.e. ANC).

### Serum APRIL levels

In contrast to BAFF, APRIL values did not change over LON time (Supplementary Table 2). There were no significant correlations between APRIL levels, on one hand, and, on the other, BAFF, CRP or AMC.

### Serum G-CSF levels

G-CSF values were significantly higher at start of LON than in post-LON samples (*p* = 0.002) and when compared with non-LON controls (*p* = 0.0004). Post-LON values did not differ from those of non-LON controls (Fig. 3D). G-CSF values at LON start showed a trend towards a negative correlation to duration of LON, i.e. those with the highest G-CSF values at start of LON tended to have the shortest LON duration (Fig. 2D).

Considering data indicating a relationship between PB levels of G-CSF and infection/inflammation in chronic autoimmune NP individuals (but not to grade of NP) [22], we asked if similar relations might be found in the acute agranulocytosis of this study. There was a trendwise significant positive correlation (*p* = 0.053) between the PB levels of CRP and G-CSF at LON start, and a highly significant correlation between CRP at LON start and the G-CSF levels at end of LON (*p* = 0.002; Fig. 5C). Moreover, raises of ANC from nadir to post-LON correlated significantly to reductions of G-CSF levels over the LON period (*p* = 0.014, Fig. 5D). Thus, during the LON period significant interactions of NP, inflammation and G-CSF levels were detected.

Next, since G-CSF is reported to be a strong inducer of BAFF secretion [8–10, 15], we looked for relations of G-CSF and BAFF at start, end of and changes over LON period, but found no significant ones (*P* > 0.05, data not shown). Likewise, no correlation was found for G-CSF levels and CD33^+^ or CD117^+^ cells at LON start (data not shown). Thus, the role of G-CSF as inducer of BAFF secretion in an acute NP such as LON remains elusive, but may depend on different kinetics of changes.

### Plasma SDF-1 values

PB SDF1 values did not differ significantly during the LON period (Supplementary Table 2); thus, there was no indication of a systemic effect on release (or re-entry) of neutrophils over the blood-BM barrier.

### Anti-neutrophil antibodies and LGL cells

Since BAFF rises have been associated with emergence of autoimmune phenomena [7] we analysed if LON associated BAFF rises might trigger development of autoantibodies to neutrophils, but none of the LON patients or the matched non-LON patients displayed GAT, GIFT or MAIGA positivity. Then, we asked if emergence of LGL-cells, previously considered to be part of the autoimmune spectrum of NPs [23, 24], related to BAFF rises at start of LON. A significant positive correlation (*P* = 0.012; Supplementary Fig. 1) was found. Thus, BAFF may play a role for the appearance of LGL-cells in LON.

## Discussion

LON is a unique form of drug-induced neutropenia (DINP) since it may start 1–12 months after last administration of the drug, whereas other DINPs start during or up to one week after treatment [25]. The reported incidence of rituximab-induced LON (5–27%) is also manifold higher than for traditional DINPs, (0.2–2% of treated patients) [26, 27]. LON represents a unique opportunity to analyze mechanisms for one form of DINP.

Our major findings of LON characteristics agree with previous descriptions [1–7, 19]. Three novel observation were that (1) the longer time to LON presentation, the shorter was LON duration, that (2) BAFF and G-CSF increases was found at LON onset, and (3) LON duration was shorter in those with the highest G-CSF levels. The reason for these phenomena is not known but may be related to genetic factors [13, 14] and feedback regulatory systems.

One unresolved question is if all rituximab-treated patients develop LON, but that those cases with a mild clinical course escape detection, implying that LON is an integral part of (side) effects of rituximab and not an idiosyncratic reaction. Our major argument against this hypothesis is that no NP cases were identified among the well-matched controls. However, to prove or reject the hypothesis of LON being an integral part of rituximab treatment requires even more frequent PB samplings than we obtained. Thus, we can, but not unequivocally so, argue that LON appears to be an idiosyncratic reaction.

The mechanisms for LON still remain elusive. Here, we focused primarily on cytokines that might be parts of the process, alone or in consort.

One previous study suggested that perturbation of SDF-1/CXCL12 during B-lymphocyte recovery retards neutrophil egress from the BM [11]and thus causes NP. We did not find any significant differences in PB SDF-1 levels here, making such an explanation less likely. Moreover, the paucity of BM mature neutrophils in LON speaks against such effect [28].

A role for NK cells in NP has been discussed [29] and may be a mechanism for LON. However, we found no changes of NK cell counts (but of CD3 + 56 + cells, i.e. T/NK cells). This issue needs phenotyping of NK cells to be resolved. Interestingly, BAFF levels at LON start correlated with frequencies of these CD3 + 56 + cells (Fig. 5B). The somewhat higher PB LGL counts in our LON patients remain elusive for the LON process but may be part of a BAFF-related mechanism also involving Fas/Fas ligand. Our finding agrees with other studies [23, 24].

A suggested deficiency of the major growth factor for neutrophils, G-CSF [15, 30], was not found here; instead, we noted rises at LON onset and reductions to levels of controls in post-LON samples (Fig. 3D). Moreover, several statistically significant correlations were found between G-CSF changes, on the one hand, and of CRP and of ANC, on the other. Based on this and data from G-CSF variations during cyclic NP and in autoimmune NP [22], we suggest that G-CSF rises observed here can be reactions to concomitant neutropenic infections as well as feed-back reactions to the abruptly emerging agranulocytosis. Since these phenomena are highly interrelated, experimental systems are needed for dissecting the chain of events. However, we cannot rule out a temporary absolute and/or functional deficiency of the cognate receptor for G-CSF, CSF3R, due to paucity of neutrophil precursors in the BM [28] that might reduce cellular uptake of G-CSF and thus accumulation of G-CSF in the PB.

Since BAFF has been pointed out as a possible mechanism for LON [4, 6, 17], we evaluated this hypothesis. Some findings here support a role of BAFF in the LON process. The first is the high BP BAFF levels at LON onset and subsequent reductions within 2–3 weeks, when ANCs are normalized. A second reason is that high G-CSF levels might suppress BAFF production at later stages of LON, which might be reflected here in the lower BAFF levels (than in controls) in post-LON samples [30]. However, there are confounding observations. One is that we did not find statistical correlations of BAFF levels to the degree of NP (i.e. ANCs), G-CSF or inflammation (i.e. CRP) levels. Another is that BAFF levels peaked at time of the lowest ANC, and lowest BAFF levels occurred at resolution of LON. These findings apparently contradict PB neutrophils as major BAFF producers in LON [8, 9]. In addition, LON BMs show a maturation arrest at the (pro)myelocyte stage of granulopoiesis [28], indicating not only PB but also BM neutrophil reductions.

The positive correlation of BAFF levels to PB AMC suggests a role for monocyte series cells as producers of BAFF, as suggested [7–9]. Despite no significant change in the AMC during LON, PB AMCs may not reflect the total body monocyte/macrophage pool size. Our data may suggest a relation between the acute NP, monocytic cells and regulation of BAFF generation. Although we did not note a significant correlation between changes of BAFF and G-CSF, other studies are needed to find if BAFF rises are secondary to emergency-induced G-CSF secretion [9].

The reason for the discordant reactions of BAFF and APRIL during LON remains to be revealed but has been observed previously [4, 6].

The propensity of high BAFF production to enhance autoimmune processes [5, 7] made us look at presence of anti-neutrophil antibodies. Such antibodies were previously suggested to be a mechanism for LON [1], but none of the patients here had detectable levels.

A weakness of our study is the rather few LON cases. However, LON is a rare side-effect and our prospective material is among the larger published so far. Another weakness is that statistical correlations for data over time may be negative because of different kinetics of changes.

One strength is that we have prospectively matched controls (for diagnoses etc.) for comparisons at similar time points as when LON developed. Another is PB and BM sampling within days after start of LON and that the same persons were followed over time.

In conclusion, our current investigations provide evidence for a complex interaction of myelo- and lymphopoiesis and suggest that a burst of BAFF PB levels may be associated with the development of LON although it is hard to distinguish between a causal and a correlative relationship. Moreover, we confirm in the LON subjects that inflammation and the acute NP is driving G-CSF rises. However, there is a need for larger patient materials as well as experimental studies to validate this model of a DINP.

## Supplementary Information

Below is the link to the electronic supplementary material.

Supplementary file1 (DOCX 29 kb)

## Data Availability

Yes.
