# Supplementary material for: The role of BAFF and G-CSF for rituximab-induced late-onset neutropenia (LON) in lymphomas
Source: Med Oncol. 2021 May 18;38(6):70. doi: 10.1007/s12032-021-01516-8 (PMC8131291; doi:10.1007/s12032-021-01516-8)
Supplement: Supplementary file 1 — Supplementary file1 (DOCX 29 kb) [file 12032_2021_1516_MOESM1_ESM.docx]

**Supplementary material**

**Methods**

*Patients*

We included prospectively 174 consecutive adult NHL patients, treated with rituximab during the period of April 2009 until March 2011 at the Hematology Center, Karolinska University Hospital, Huddinge, Stockholm, Sweden.

*Patients* were treated and followed-up according to standard care protocol at the discretion of the treating physician, however, all received rituximab. The following entities were included: diffuse large B-cell lymphoma (DLBCL, n=103), follicular lymphoma (FL, n=31), mantle cell lymphoma (MCL, n=14), chronic lymphatic leukemia (CLL, n=12), marginal zone lymphoma (MZL, n=9) and lymphoplasmacytic lymphoma (Morbus Waldenström (MbW, n=5)(Figure 1). Median age was 62 years (range, 26-83). Fifty-three percent (n=90) were males and 47% (n=79) were females. Seventeen patients were treated with rituximab alone, at 375 mg/m^2^. The remaining patients received rituximab, at the same dose, in combination with chemotherapy (except in CLL, where rituximab was administered at 500 mg/m^2^ starting from the 2^nd^ cycle of chemotherapy); details are given in Supplementary Table 1. CBCs (complete blood counts) were obtained routinely every fortnight to month.

*Exclusions* Medical records were reviewed for the time of two years before the start of rituximab therapy and all patients with a previous history of neutropenia were excluded. Thus, one patient with MbW was excluded because of a previous history of autoimmune cytopenia. Two patients with DLBCL, treated with neutropenia-causing drugs, and one patient with FL, experiencing a short-term infection deemed to be viral, were also excluded. One MZL patient was lost to follow-up. Hence, 169 patients were included for LON analysis (Figure 1).

*Controls* A control group within the same cohort of NHL patients was established simultaneously with the detection of a LON episode. This comparison was done to control for confounding factors for the neutropenia. Initially, two control patients were recruited for each LON case among patients undergoing lymphoma treatment at the same time as the LON patients. They were matched by age, sex, diagnosis, stage of the disease, treatment (intensity, type, de novo or relapse). Total dose of rituximab (and methotrexate) given under the whole course of chemotherapy was also matched. The paucity of patients with MCL, and unforeseeable events after selection, reduced the number of controls to 26 subjects (Table 1). In addition, a subgroup consisted of 10 of these 26 patients who volunteered to provide BM samples in addition to PB samples.

*Definition of LON* LON is defined as an otherwise unexplained PB absolute neutrophil count (ANC) of ≤ 0.5 G/L (corresponding to grade 4 neutropenia according to National Cancer Institute Common Toxicity Criteria (NCI-CTC)^18^ and agranulocytosis as ANC ≤0.1 G/L, starting earliest four weeks after the termination of rituximab therapy. Absolute PB monocyte counts (AMC) were considered normal if 0.2-0.8 G/L. All patients were followed for, at least, 12 months after rituximab treatment. This concept was based on the previous findings of a sustained B-lymphocyte depletion for up to one year in rituximab-treated patients.^19^ Four patients had undergone an autologous HSCT as part of previous therapy for the lymphoma (Supplementary Table 1).

*Peripheral blood (PB) and bone marrow (BM) evaluations*

In LON patients, PB samples were obtained at two occasions: first, as soon as possible at detection of LON (“LON samples”) and, second, after resolution of LON (“post-LON samples”); they were stored at -70°C for further analysis. BM samples were obtained at the first PB sampling and were processed according to clinical routines. In addition, we retrieved routine clinical PB ANC and AMC immediately preceding onset of a LON episode (i.e.1/-2/ weeks). In patients given G-CSF due to LON, BM and PB samples were collected before the initiation of G-CSF treatment (except for 1 case) and, at least, one week after stopping that therapy (except for 2 cases); all data are shown for those without ongoing G-CSF treatment. PB samples were collected only at the first episode of LON in cases of repeated neutropenia episodes during the follow-up period. LON and post-LON PB samples were also used for serology and serum cytokine tests. A detailed medical history and physical examination was performed at LON to rule out alternative causes of NP.

Relative LGL cell abundance was analyzed by light microscopy on routinely stained PB smears, as advised by Loughran & Lamy.^20^.

In the matched controls, BM (n=10) and PB samples (n=20) were also collected at time points corresponding to the detection of LON in the LON patients, in order to control for potential time-related effects for BM regeneration after the last rituximab infusion and chemotherapy; they were stored or processed as for LON samples.

*BM flow cytometry* was performed by using four or eight color fluorescence immunophenotyping for B and T lymphocytes, and for natural killer (NK) cells, as previously described.^14,19^ For analysis of B-cells, fluorochrome-conjugated monoclonal antibodies to B-cells antigens CD19 and CD20 were used. For analysis of T-cells and NK-cells, anti-CD3, anti-CD4, anti-CD8, anti-CD16 and anti-CD56 antibodies were used. Analyses concerning myelopoiesis will be reported separately. All antibodies were obtained from Becton Dickinson (Mount View, CA, USA). Data were acquired and analyzed with a FACSCantoII and FACSDiva software (Becton Dickinson). All samples were analyzed by setting appropriate side and forward scatter gates. Consistency of analysis parameter was ascertained by calibrating the flow cytometry with calibrating beads and FacsComp software, both from Becton Dickinson. For the cell subpopulation analysis, a minimum of 10 000 events were collected in the gate.

B cell depletion (analyzed by flow cytometry) was analyzed as percentage of BM cells expressing CD20.

C-reactive protein (CRP) PB levels were analyzed according to routine procedures at the Karolinska University Laboratory.

*Enzyme linked immunosorbent assay (ELISA)*

Serum levels of human BAFF, APRIL, G-CSF and plasma levels of SDF-1 (a k a CXCL12) were analyzed by using Quantikine immunoassays (R&D Systems Europe) according to the manufacturer’s instructions. All assays specificity and reproducibility were ascertained by the manufacturer. All samples were analyzed in duplicates.

*Anti-neutrophil antibodies*

Tests for anti-neutrophil antibodies were performed at a national certified laboratory (Department of Laboratory Medicine, Division of Clinical Immunology and Transfusion Medicine, Karolinska University Hospital Huddinge). The initial test panel included granulocyte agglutination test (GAT) and granulocyte immunofluorescence tests (GIFT). Complementary analysis by monoclonal antibody-specific immobilization of granulocyte antigen (MAIGA) test^21^ was used in positive cases to characterize the specificity and to rule out false positivity. All samples were also analyzed for HLA antibodies which can induce false positive GAT or GIFT; if such antibodies were detected a MAIGA test was run.^21^

**Results**

The median time to onset of LON was 96 days (Figure 2A; range, 41-230). The median duration of LON was 17 days (Fig. 2B; range, 7-33). Four were febrile and 2 reported feelings of fever (but had normal CRP). Nine patients received treatment with G-CSF (Figure 2A,B). The median nadir ANC was 0.2 G/L (range, 0.1-0.5); thus, all LON patients developed severe neutropenia (Figure 3A). The 20 non-LON controls, with available CBC corresponding to the time to LON of their matched pairs, displayed significantly higher ANC and none displayed ANC<1.5 G/L (P<0.0001; Figure 3A). PB absolute monocyte counts (AMC; Figure 3B) for LON patients were similar to those of non-LON controls (P>0.05 for all comparisons; Figure 3B) and did not correlate to change of ANCs (P>0.05); thus, those with the most pronounced ANC drop did not raise their AMC more than those with least pronounced ANC drops.

Time to LON and LON duration correlated significantly, in that those with a short time to LON had longer duration of LON than those with a long time to LON (P=0.048; Figure 2C). LON-patients with a previous HSCT (n=4; 27% of all LON patients) had similar incidences and complications to LON as those without (Figure 2A, B).

LON emerged suddenly in most patients, often within one week; a few displayed a more protracted course of onset of LON over 2-3 weeks. The median time to onset of LON was 96 days (Figure 2A; range, 41-230). The median duration of LON was 17 days (Figure 2B; range, 7-33). Nine patients received treatment with G-CSF (Figure 2A,B). The median nadir ANC was 0.2 G/L (range, 0.1-0.5). Thus, all LON patients developed severe neutropenia (Figure 3A). The 20 non-LON controls, with available CBC corresponding to the time to LON of their matched pairs, displayed significantly higher ANC and none displayed ANC<1.5 G/L (P<0.0001; Figure 3A). PB absolute monocyte counts (AMC; Figure 3B) for LON patients were similar to those of non-LON controls (P>0.05 for all comparisons; Figure 3B) and did not correlate to change of ANCs (P>0.05); thus, those with the most pronounced ANC drop did not raise their AMC more than those with least pronounced ANC drops.

There was no concurrent reduction in PB platelet or erythrocyte counts during the LON period.

Time to LON and LON duration correlated significantly, in that those with a short time to LON had longer duration of LON than those with a long time to LON (P=0.048; Figure 2C). LON-patients with a previous HSCT (n=4; 27% of all LON patients) had similar incidences and complications to LON as those without (Figure 2A, B).

Six patients were admitted to the hospital, receiving *inter alia* parenteral antibiotics. None died during the LON period or shortly afterwards. A total of 11/15 patients were given filgrastim treatment.

**Supplementary Table 1.** LON patient characteristics, treatments, bone marrow and clinical features.

Pt Diagnosis Age Ann NHL Treatment Days to ANC Filgrastim/

no (y/sex) Arbor onset/duration nadir, antibiotics

stage of LON G/L treatment

1 DLBCL 74 /F II 8 x R-CHOP + RT 135/14 0.2 Yes/Yes

2 MCL 71/M III 3 x R-Maxi CHOP 96/7 0.1 No/No

3 x R-HIDAC + HSCT

3 MCL 68/F IV 3 x R-Maxi CHOP 81/12 0.2 No/No

3 x R-HIDAC + HSCT

4 FL 63/F IV 2 x R-DHAP 118/12 0.1 Yes/Yes

2 x R-HIDAC + HSCT

5 CLL 62 /F IV 5 x R-FC 113/24 0.3 No/No

6 FL 60 /F IV 6 x R-CHOP 45/15 0.1 Yes/Yes

7 CLL 59/M IV 6 x R-FC 95/8 0.3 Yes/No

8 DLBCL 57/M III 6 x R-CHOEP 230/8 0.1 Yes/No

9 DLBCL 45 /M 6 x R-CHOEP 129/14 0.1 Yes/Yes

10 FL 55/M II 4 x R-MIME 89/21 0.1 Yes/No

11 DLBCL 52/F IV 2 x R-DHAP + R-MTX 58/20 0.1 No/No

2 x R-HIDAC + HSCT

12 DLBCL 50/M III 6 x R-CHOP 140/15 0.3 Yes/No

13 MZL 46/M IV 6 x R-FC 104/27 0.4 Yes/No

14 FL 36/M III 6 x R-CHOP + 49/33 0.5 No/No

R-maintenance

15 DLBCL 35/F IV 6 x R-CHOP + RT 41/29 0.4 No/No

ANC nadir, the lowest absolute neutrophil count; BM, bone marrow; CHOP, cyclophosphamide, doxorubicin, vincristine, prednisolone; CHOEP, cyclophosphamide, doxorubicin, vincristine, etoposide, prednisolone; CLL, chronic lymphatic leukemia; DHAP, dexamethasone, cisplatin, cytarabine, prednisolone; DLBCL, diffuse large B-cell lymphoma; FC, fludarabine, cyclophos-phamide; F, female; FL, follicular lymphoma; HIDAC, high dose cytarabine; M, male; MZL, marginal zone lymphoma; MCL, mantle cell lymphoma; MIME, metyl-gag, holoxan, uromitexane, methotrexate, vepeside; NEv, not evaluable due to PB admixture in the aspirate; R, rituximab; RT, radio therapy; HSCT, autologous hematopoietic stem cell transplantation.

**Supplementary Table 2.** Peripheral blood values for APRIL and SDF-1 (CXCL12).

At LON Post-LON Controls

­­­­­­­­­­­­­­­­­­­­________________________________________________________________

APRIL, ng/mL 3.38 ±5.44 4.70±6.84 4.70±7.43

SDF-1, ng/mL 2.01±0.34 2.05±0.41 2.03±0.50

Mean±SD values. None of the differences (LON vs Post-LON, LON vs Controls, or Post-LON vs Controls reached statistical significance (P>0.05).

**Supplementary Figure 1.** Spearman’s correlation between peripheral blood concentrations of BAFF and percentage of LGL cells, both at start of the LON period. The dotted lines depict the 95% confidence interval for the regression (solid line)
